# Supervisory knowing in practice across medical specialities

**DOI:** 10.1007/s10459-023-10251-w

**Published:** 2023-06-13

**Authors:** Christy Noble, Joanne Hilder, Stephen Billett, Andrew Teodorczuk, Rola Ajjawi

**Affiliations:** 1 Academy for Medical Education, Medical School, The University of Queensland, Herston, Queensland Australia; 2 Department of Allied Health Services, Gold Coast University Hospital, Gold Coast Hospital and Health Service, Southport, QLD Australia; 3 School of Education and Professional Studies, Griffith University, Brisbane, Queensland Australia; 4 The Prince Charles Hospital, Metro North Mental Health, Brisbane, Queensland Australia; 5 School of Medicine and Dentistry, Griffith University, Southport, Queensland Australia; 6 Centre for Research in Assessment and Digital Learning, Deakin University, Melbourne, Victoria Australia

**Keywords:** Clinical supervision, Workplace learning, Practice theory, Metaphor, Specialities

## Abstract

Clinical supervisors play key roles in facilitating trainee learning. Yet combining that role with patient care complicates both roles. So, we need to know how both roles can effectively co-occur. When facilitating their trainees’ learning through practice, supervisors draw on their skills - clinical and supervisory - and available opportunities in their practice. This process can be conceptualised as supervisory knowing in practice (or contextual knowing) and offers ways to elaborate on how facilitating trainees’ learning can be optimised. The practice-based study presented and discussed here examined clinical supervisors’ knowing in practice related to facilitating trainee learning, across three medical specialities. Nineteen clinical supervisors from emergency medicine, internal medicine and surgery, were interviewed about their roles and engagement with trainees. Interview transcripts were analysed in two stages. Firstly, a framework analysis, informed by interdependent learning theory was conducted, focussing on affordances and individual engagement. Secondly, drawing on practice theory, a further layer of analysis was undertaken interrogating supervisors’ knowing in practice. We identified two common domains of supervisor practice used to facilitate trainee learning: (1) orientating and assessing trainees’ readiness (or capabilities), (2) sequencing and enriching pedagogic practices. Yet across the speciality groups the supervisors’ knowing in practice differed and were shaped by a trio of: (i) disciplinary practices, (ii) situational requirements and (iii) clinician preference. Overall, we offer a new reading of clinical supervision as practice differences generated distinct supervisory knowing in practice. These findings emphasise clinical supervision as fundamentally entwined in the speciality’s practice; and reinforce alignments with patient care.

## Introduction

Medical trainees develop their clinical capacities largely by learning through practice (Dornan, 2012; Teunissen, 2015). Clinical supervisors play an essential role in enhancing trainees’ learning in this practice-based mode of learning in clinical settings. Consequently, the processes of clinical supervision and clinical teaching have been widely examined. What remains unclear is how supervisors facilitate trainees’ learning whilst engaging them in clinical practice and fulfilling their role as clinicians. In other words, how do clinical supervisors accommodate the circumstances of practice (i.e. disruptions and affordances) as they facilitate trainee learning. This question purposefully disregards the opposition often created between patient care and trainee learning to focus on how learning can best co-occur with clinical practice. So, there is a need to understand how supervisory knowing is manifested in the circumstances of the actual practice, rather than abstracted notions of required or idealised supervisory practices. Such an approach might enable more sustainable enactments of clinical supervision that are embedded within and responsive to the circumstances of practice. Our key research questions are: How are clinically-embedded supervisory practices manifested across different specialties? How might this understanding help us to reconceptualise clinical supervision?

There is a rich seam of research into clinical supervision and, over time, understandings about it have changed. For instance, conceptualisations of effective supervisory practices have shifted from being largely teaching-focussed approaches (e.g. teaching on a ward round (Ker, et al., 2008)) towards more learner-centred approaches (Dornan, 2006; Pront, et al., 2016). Clinical supervision is also now more broadly understood as facilitating trainee learning in the workplace (Dornan, 2006, 2012) where pedagogical strategies include guidance and support (noting that pedagogical strategies might well include ‘teaching’ but not exclusively). These conceptual shifts are being acknowledged in frameworks outlining clinical supervisory skills (e.g. the Academy of Medical Educators domains (The Academy for Medical Educators, 2023). Faculty development programs have now been revised to educate supervisors about facilitating trainee learning (Steinert, 2014). Despite these advances, individual supervisors are left to make decisions about how they will best facilitate trainee learning in practice whilst accounting for the opportunities and constraints of clinical environment (Cantillon, et al., 2020). As a result, considerable variation exists in how clinical supervisors make decisions and enact their supervisory skills. Consequently, the enactment of supervision is often based on individual preferences, rather than evidence informed practice (Goldszmidt, et al., 2015). ‘Excellent’ supervisors employ strategies to sequence and select learning activities within the workplace based on learner readiness (Chen, et al., 2015; Steinert, et al., 2017). However, it is acknowledged that the demands of the clinical work and environments (i.e. situational factors) shape supervisory practices (Chen, et al., 2016; Steinert, et al., 2017). Supervisors respond to these demands by employing different kinds of pedagogical strategies (e.g. questioning, discussion)(Steinert, et al., 2017). Invariably though, it is reported that heavy workloads or juggling multiple tasks, make the enactment of facilitating trainee learning challenging because of the pressures of clinical care that are often described as obstacles to effective learning facilitation by supervisors (Sholl, et al., 2017). A key challenge described by clinical supervisors is that the imperatives of patient care override the facilitation of trainee learning. Indeed, the prevailing metaphors related to clinical supervision is the balancing act between patient care and facilitating learning - when the balance is, understandably, tipped towards patient care, learning facilitation is set aside (Dennis, et al., 2014; Sholl, et al., 2017). Pitching facilitation of learning in opposition to patient care will likely be more detrimental to trainee learning because the realities of practice will always make it challenging for supervisors to enact their supervisory skills. However, embedding that supervision within clinical practice may offer a means of achieving both goals.

Indeed, research has begun to dispel the somewhat acontextual conceptualisations of clinical supervision. For instance, Cantillon et al. (2020) proposed that working, learning and clinical supervision are interlinked and deeply contextual practices. They found that clinical supervisors’ roles are shaped by everyday practices including the dynamics of medical team structures and relationships. For example, learner and supervisor roles are co-constructed through dynamic interplay with the team’s implicit curriculum (including norms, standards and expectations). Moreover, further work by Cantillon et al. (2022) emphasises the “profoundly situated nature of the curriculum of the workplace, and the particularities of teaching in different specialities” (p.622). Whilst exploring learner and teacher (i.e. supervisor) identity formation, they also identified unique ways of knowing, being and conversing exist within different specialities (i.e. internal medicine and surgery) and these shaped ways of learning and supervising. Overall, Cantillon et al. (2022) have highlighted that “the associated practices of teaching and learning are all shaped by, and contingent on, the sociocultural contexts in which clinical learning is situated”(p.622). Their findings prompt further work to explore supervisory ways of knowing in differing circumstances of clinical practice. Further it remains unclear how supervisory practice associated with facilitating trainee learning are generated. By illuminating and elaborating how supervisors mobilise knowledge in practice, we can better account for effective practices in supporting clinical supervision, rather than separating the two.

Overall, the ongoing challenges suggest that the realities and complexity of supervising in clinical practice have not yet been fully elaborated. What is remains unexplored is how clinical supervisors from diverse disciplines enact their supervisory skills within the realities of clinical practice, thereby emphasising close links between clinical practice and effective supervision. Rather than trying to circumvent practice challenges by identifying incongruencies between patient care and clinical supervision or attempting to resolve these tensions, we need to remove and reconcile distinctions between clinical supervision and patient care. To redress this gap, we explored clinical supervisors’ knowing in practice to better understand how they facilitate trainees’ learning in practice. From the perspective of knowing in practice, the supervisory practice is not simply an application of acquired knowledge (i.e. conceptual knowledge about supervision). Instead, supervisors draw on sets of clinical knowledges as resources for action, and through this act, supervision produces further knowledges (Gherardi, 2019, p. 53). Indeed, knowledge itself does not reside within the individual rather it is manifested, and shaped by, particular circumstances of the practices (Billett, 2001; Gherardi & Nicolini, 2006).

### Workplace learning – theoretical perspectives

To illuminate and elaborate how supervisors’ knowledge is manifested as they facilitate trainees’ learning whilst accounting for the circumstances of practice, two complementary theoretical perspectives are instructive: 1) interdependent learning (Billett, 2006) and 2) knowing in practice (Billett, 2001; Gherardi & Nicolini, 2006). Firstly, to understand supervisors’ learning facilitation, the theorization of learning through practice was adopted. From this perspective, learning is theorized as an interdependent process between learners’ individual agency (e.g. intentionality, readiness, subjectivity and identity) and workplace affordances (e.g. workplace norms, practices and values) (Billett, 2006). The key premise is that neither social experiences and practices (e.g. clinical teaching, ward rounds or surgical operations) nor individuals’ (e.g. trainees’ readiness (e.g. what they know, can do and value) alone is sufficient to effect learning through practice. Rather, the interdependence between those affordances and engagement is essential. This perspective of workplace learning is important because, whilst it acknowledges that learning occurs through teaching in clinical settings, it includes the range of clinical activities and interactions in which trainees engage, and a broad repertoire of strategies supervisors can employ that liberates clinical teaching from being restricted to just telling. These strategies (e.g. providing guidance, heuristics, sharing stories) are often both shaped by and augment the learning through the practice situation, referred to as practice pedagogies (Billett, 2016). Furthermore, there is an interdependence between the strategies afforded trainees and the degree by which the trainees take up the invitation to engage with these strategies. Hence, the learning process occurs through a dynamic interplay between learners’ engagement in the activities and interactions afforded by clinical practice, including the actions of supervisors.

While interdependent learning theory gives us a dialectic understanding of learners always being in relation with the workplace – the supervisors’ knowing in facilitating the dialectic is elided. Practice theory also assists here. In particular the theoretical work of Gherardi (2019) helps with conceptualising knowing (i.e. supervisory knowing) as a situated activity – a knowing-in-practice – whereby “knowledge emerges from the context of its production and is anchored by (and in) material supports in that context” (Gherardi, 2019, p. 50). The situatedness of activity goes beyond skillfulness related to that activity (in our case, the supervisory skills required to facilitating trainee learning), but of necessity incorporates situational factors (e.g. workload; team members) that shape how supervisors’ knowledge and skills are manifested in particular circumstances of practice (Gherardi, 2019). It follows that supervisory knowing in practice is not fixed rather it is enacted in and through particular circumstances of practice.

Moreover, when conceptualising the circumstances of practice, Gherardi (2019) describes them as “a texture of practices” (Gherardi, 2019, p. 24). This conceptualization acknowledges the rich, dynamic, interconnected and complex nature of practice. Thus, the textures of supervisory practices in specific clinical settings and particular clinical specialties (e.g. medicine and surgery) will differ. To better understand knowing in practice, Gherardi quoting Henion introduces the metaphor or rock climbing to instantiate how “practice emerges and is socially and materially sustained” (Gherardi, 2019, p. 50). This metaphor has been explanatory for elaborating on work integrated learning (Dean & Sykes, 2021). In this metaphor, the rock face represents practice and the knowing in practice is represented by climbing (e.g. knowing how to read the rock face and seeing handholds along the way to inform the climbers next move) (Gherardi, 2019). In our case, climbing represents supervisors’ knowing in practice and the handholds represent the support and guidance they create to facilitate trainee learning, whilst the rock face is represented by particular circumstances of practice. In sum, together the two theoretical perspectives assisted addressing the research questions. Our research questions are: *How are clinically-embedded supervisory practices manifested across different specialties? How might this understanding help us to reconceptualise clinical supervision?*

## Methods

This practice-based inquiry (Gherardi, 2019) adopts a social constructivist approach, shaped specifically by cultural psychological (Billett, 2006) and practice theory (Gherardi, 2019) perspectives that hold knowledge to be subjectivist, relational and situated. Ethical approval for the research was obtained from Gold Coast Health ethics committee: HREC/18/QGC/32.

### Recruitment and sampling

Purposive and snowballing methods were used to recruit supervisors. Senior clinicians working in emergency medicine, internal medicine and surgery in a large public sector Australian tertiary teaching hospital were invited to be interviewed for this study. Senior clinicians are defined as those in either consultant roles (i.e. completed all their speciality training) or advanced trainee roles (i.e. completed their speciality examinations with one or two more years of clinical training remaining) and contributed to trainee learning. Trainees were defined as medical students to junior doctors with up to five years’ experience. Three distinct clinical specialties were selected to enable a comparison across different circumstances of practice. All consultants and advanced trainees in each speciality being explored were invited to participate in the study. The email invitations were distributed by the department heads. Respondents were sampled for diversity in relation to gender, seniority of doctor level and specialty.

### Data collection

Having gained informed consent, 19 semi-structured interviews were conducted with senior clinicians. The interview schedule was informed by interdependent learning theory (Billett, 2006). and explored how senior doctors engage with and work with trainees (as per description above) to facilitate their learning. The interviews were audio recorded, deidentified and transcribed verbatim. Whilst there are limitations of relying on interviews alone for unpacking practice, open and grounded interviews of the kind adopted and enacted here offer windows into practices (Cameron, et al., 2019; Gherardi, 2019), and, therefore, are commonly enacted in practice research (Bearman, et al., 2021). To secure insights into practice, we asked about actual events, healthcare team structures, and how they engaged with and work with trainees. The interview guide is provided in the supplementary material for reference. We continued interviewing until team members agreed that the data gathered sufficiently answered our research question. Sufficiency was defined as having enough data to have a good enough understanding of supervisors’ knowing in practice across the three specialities (Varpio, et al., 2017).

### Data analysis

The data were firstly analysed through a five stages: (1) familiarisation; (2) identifying a thematic framework (informed by interdependent learning theory); (3) indexing; (4) charting and (5) mapping and interpretation (Ritchie & Spencer, 1994). Practically, interviews were transcribed and shared with all research team members. A subset of three transcripts was read by the team and discussed with interdependent learning theory as a sensitizing lens to identify the domains of supervisory practice in the workplace (Billett, 2006). Based on this inductive and deductive coding and through further discussion, a preliminary coding framework was developed. The entire dataset was then uploaded to NVivo® and analysed using this framework. Through these preliminary and subsidiary analyses, we identified common domains of supervisory practices within the supervisors’ accounts of facilitating trainee learning. These domains of supervisory practice were identified using interdependent learning theory. Yet, what remained unexplained was the differences between the specialities nor were we able to position the supervisor easily within our explanations. We needed an analytical approach that allowed for a more holistic account of how supervisors knowledge is manifested through practice as they facilitate trainee learning. To better understand supervisory knowing in practice we engaged in a second phase of data analysis.

The second level of data analysis was interpretive; informed by Gherardi’s practice theory (Gherardi, 2001, 2016, 2019). Team members read and re-read the data pertaining to facilitating learning, sharing interpretations of how supervisors’ knowledge was manifested in their practice, what these practices were and how they differed across specialties and setting to illuminate supervisory knowing in practice. We used the rock-climbing metaphor previously introduced as a sensitising lens to offer an alternate reading of clinical supervision.

### Reflexivity

Our multi-disciplinary research team included two health care professionals with PhDs in health professions education and established programs of research in workplace learning (RA and CN); one educationalist with PhD and extensive programs of research in workplace learning including pioneering interdependent learning theory (SB); one senior staff specialist with MD in medical education (AT); and one PhD candidate with a social work background (JH). Through regular discussions we drew on our specific perspectives to analyse the supervisors’ accounts. For example, the clinicians elaborated on supervisory practices whilst other members illuminated theoretical explanations.

## Results

As noted, we interviewed 19 people from across three specialties: (i) emergency medicine (n = 8), (ii) internal medicine (n = 6) and (iii) surgery (n = 5). The interviews ranged from 26 to 81 min with the median being 45 min. The analysis of the interview data identified that supervisors drew on common supervisory practice to facilitate trainee learning across two domains of practice: (1) orientating and assessing trainees’ readiness, (2) sequencing and enriching pedagogic practices. Beyond these common approaches, the supervisors’ knowing in practice was shaped by a trio of: (i) disciplinary specific practices, (ii) situational requirements and (iii) individual preferences. The results section, begins with an elaboration on these two domains of common supervisory practices whilst highlighting features of supervisors’ knowing in practice within their specialities (i.e., commonalities and differences). To conclude, the rock-climbing metaphor is used to further elaborate supervisory knowing in practice.

1. Domain of supervisory practice - Orientating and assessing trainees’ readiness to practice.

The supervisors emphasised the importance of orientating their trainees and assessing their readiness to learn through practice. Yet the supervisors’ knowing in practice emerged differently as they enacted this practice in their medical specialism. We found that, in particular, the different supervisory practices were influenced by the duration and nature of co-working.

In terms of duration, for both medicine and surgery supervisors’ co-working with their trainees was sustained over several weeks (e.g., 5 to 12 weeks) and within small teams (e.g., registrar, residents and medical students). The supervisors knew who and when trainees were coming to their department and could purposely orientate and help trainees to adjust to the requirements of the clinical practice. Firstly, the supervisors initially oriented trainees situationally (i.e. physical and social setting); and discussed the tasks trainees needed to perform. They explained how their team worked and its communication practices (e.g. “I’ll tell them, call your registrar first” (P2 Medicine); “give them my phone number so they often will just text me throughout the day” (P4 Medicine)).

Secondly, supervisors in medicine and surgery often reported seeking to understand their trainees’ career goals and them as a individuals e.g. “I’m asking them where they come from, where is their family, are they married, what pets they have, if they have kids” (P2 Medicine). A surgeon, who conducted operations the day trainees arrived, preferred to establish rapport prior to the trainees’ first day by hosting a social function at their house:

… just for a very relaxed barbecue with partners and kids and just kind of demystify the whole thing. There’s that socialisation, too, where they hand on all their tips and we share a meal together. (P6 surgery)

These types of conversations enabled supervisors to personalise supervision by: (1) making explicit connections between the practice and trainees’ career goals; (2) responding sensitively to trainees’ home situations e.g. if they need to care for sick children; or (3) smoothing trainees’ transitions (e.g. from medical school, different specialities/terms). A particular focus was facilitating trainees’ understanding that internal medicine is a team-based practice, and not a solo, act:

…sometimes it has to be pointed out to them [intern] that the competency is not only your cognitive development, it’s also your development of activity-based competency…beyond that, how to work in a team – for them to understand the infrastructure within which we work and who else is involved in caring for the patient, and in what capacity...My role is to try to point these differences to them and the human interactions between the teams – how they can be powerful in improving the patient care or how they can end up being disruptive in patient care. (P8 Medicine)

Strategies to support trainee learning were reported as being shaped by patterns of work practices. As patient-related activities presented themselves, supervisors assessed their trainees’ readiness, worked together and if trainees did not know how to perform the task, then they demonstrated it:

…the first day I’ll meet them (intern) and say, this is the team, this is how the week runs, this is what we expect you to be doing and I guess we troubleshoot as we go along. So, you might say, can you refer this patient for an echo and they’re like, I don’t know how to do that. So, you get the form and you show them how to do it, or if someone dies you show them the black box and how to fill out a death certificate and what forms they need to fill in, basic stuff like that really. (P4 Medicine)

In contrast, the Emergency Department (ED) supervisors’ co-working with trainees was shift based, short term (e.g. 10–12 hours) and tended to be dyadic (i.e. supervisor and trainee). Many ED shifts comprised working with new trainees. ED is a large department and “…there are so many interns and they don’t stay for very long” (P15 ED). Also, because ED supervisors had other pressing responsibilities e.g., managing patient flow through the department; caring for their own patients, they needed to rapidly assess their trainees’ readiness and whilst sharing their expectations on co-working. These circumstances of practice meant that the supervisors’ assessment of trainee readiness tended to be contained within a shift. The following quote illustrates this approach:

If I have met the intern for the first time then I always ask them is this your first day, week? How long have you been into ED? What other areas you’ve worked in ED. What previous rotations you’ve done. Just to give me an idea about if they had different exposure. Because if it’s their first time out of medical school into ED I think that’s, you know, they’re green, they haven’t actually had any exposure, so … that will also help me gauge and know exactly where are they at this stage. Then, of course, the work has to be done and this is a balance that we have to work with. (P7 ED)

In this instance, the supervisors’ knowing in practice emerged through working within a complex process of seeing patients, making assessments of both the patients and trainees whilst supervisors had their own shift responsibilities. Emphasised in these short shifts, compared to medicine and surgery, was the need to quickly ascertain the trainees’ readiness to engage as part of the ED team.

Across the three specialities, there were distinct situational features associated with their circumstances of practice and practitioner preferences that shaped how supervisors orientated trainees to the particular clinical practice and how trainees’ readiness was assessed, and clinical training progressed. It was evident that the duration and nature of supervisor-trainee co-working were key premises influencing supervisors’ knowing in practice, and these were shaped by the practice of the clinical community as manifested in those particular healthcare settings.

2. Domain of supervisory practice - Sequencing and enriching learning.

All supervisors described a common repertoire of broad supervisory practices to sequence and enrich trainee learning (e.g., increasingly complex tasks, bedside teaching). Yet, as with assessing trainees’ readiness, there were unique practice structures (e.g. physical environment; nature and duration of co-working) and situational factors (e.g. dominant clinical activities; workload) that shaped supervisors’ knowing in practice and how they approached facilitating trainees’ learning. For example, whilst the ward round was a common activity for each speciality, supervisors mediated trainee engagement in different ways. For medicine supervisors, ward rounds were the focal practice for facilitating trainees’ learning, and so within this practice they created sequenced activities to enrich trainee engagement. This was less so within surgery where trainees often conducted solo ward rounds, as their supervisors were in theatre, or they were brief. Instead, the dominant learning practice was in the operating theatre. Then, in ED, ward rounds occurred in some parts of ED e.g., short stay and this was enacted as a supervisor-trainee dyad (i.e., done just in time; when required). In this way, supervisory practice shaped supervisors’ knowing in practice and speciality imperatives. Moreover, factors such as longer terms and time with trainees, meant that supervisors could sequence increased trainee engagement with particular clinical tasks. This was noteworthy in ward rounds in medicine and operating in surgery.

### Emergency medicine

ED supervisors (as noted above), knowing they worked with trainees for short shifts, yet had responsibilities for patient care, enacted their supervision within a dyad (i.e. supervisor – trainee). They used specific strategies to interweave their clinical work with activities to facilitate trainees’ learning whilst responding to the ebb and flow of the department’s patient flow. They began their shift by making an initial departmental-level assessment to account for factors such as patient flow, busyness of the department and to determine what will be possible for the trainee. In some cases, at this point, they may allocate their trainee to someone else to ensure that the trainees’ learning was enriched:

… I’ll kind of make a decision slightly related to how busy it is. But, I think if it is busy I will assign them [medical student] to a registrar or a junior doctor because I think they get way more out of the day than being with me with where I am basically managing patient flow, which isn’t necessarily that relevant to a medical student. So, I think they get more out of the day there with someone who is actually doing clinical things. (P11 ED)

For supervisors who continued to work with their trainees, they explained how they like to work with and their expectations of them. These conversations set the tone and provided a mechanism for trainees to access ED practice. Learning facilitation was embedded within these expectations including allocating clinical tasks (e.g. take a history from patient x), decision making processes, and ways of communicating (e.g. when to report back to the supervisor). Trainees would then engage in their clinical tasks while the supervisors attended to their own patients and maintained patient flow. Supervisors also monitored their trainee’s progress by “[using] all your senses…you’re listening. (P3 ED)”and would check in with them e.g., “you haven’t told me about your patient yet. Tell me about them and where you’re up to (P3 ED).”

As they co-worked, the supervisors reported ensuring they were accessible to the trainees and aimed to interweave teaching moments and were creating these opportunities based on the availability of clinical activities. Some supervisors reported achieving this by checking in with the trainee and when needed providing additional guidance for learning and so the trainee can keep working:

… generally it’s about, I don’t know what to do with this patient. It would be about just exploring what they’ve done so far, what they’d like to do, and what they need to do next and why… they check in, I tell them a plan, they go away and do it … or …I tell them to go back and get some more information and then they come back and we try and make a plan. (P15 ED)

When their workload became heavier and more intense, the supervisors adjusted their learning facilitation by reducing direct teaching such as explanations and deployed strategies such as more parallel working or revisiting the distribution of tasks. Yet, the inability to directly instruct did not mean that trainees’ learning was compromised. As noted by one participant, trainee learning was ultimately enriched:

…so creating capacity…may ultimately result in…that pair [supervisor and trainee], splitting up and seeing two patients individually and then coming back. So, it might be that the patient that I see, I’d see and manage on my own and send home which I suppose can deprive that intern or JHO [junior house officer] from that learning opportunity. But then that intern or JHO can present the other case back to me and gives them, I suppose, the opportunity of, again, the learning opportunity of how do you, as a senior decision maker, go into that mindset of reviewing someone who’s already started care. (P10 ED)

For many ED supervisors, by asking trainees to perform peripheral, but informing, tasks, such as writing up medical notes and discharge summaries, created opportunities to assess their trainees’ clinical capabilities whilst easing their workload. This process entailed a process of constant negotiation and awareness of what else was occurring at the time given the dynamic nature of ED. One supervisor explains:

…we’ll see a patient and we will have a chat, and I will sort of highlight the important things in that patient. But then when they [trainee] go to write the notes it’s often a little bit - not confused, but just not prioritised, I guess. So, having that feedback is useful. Like it helps me that they’re writing the notes because then I don’t have to write, but also giving them feedback on how to effectively communicate your concern [in the notes]. (P15 ED)

As well as attempting to ensure trainees were safe, supervisors also reported needing to ensure that they, and the patients, were safe. They reported appraising whether it is safe for them respond to the trainee or whether, for patient safety, they needed to prioritise patient care. Supervisory knowing in practice emerged in offering an alternative co-working arrangement (e.g. asking someone else):

…there is always this heightened sense of arousal and tension, I suppose, when it is busy, or when you’ve got a super sick patient in the department. I think we are reasonably good at being very conscious of that. I’ll even overtly say to the juniors in the team, would you mind finding somebody else, because my head is really full at the moment and I could listen to your story but you’re going to have to tell it to me four times because I keep getting distracted? So I’ve learnt to just be very honest with when I’m feeling overwhelmed. (P16 ED)

In sum, the ED supervisors’ knowing in practice was enacted by, first, assessing the department’s workload and trainees’ readiness and, as they co-worked, adjusting their supervisory approaches in response work pressures, which may be common practice across the three areas, but manifested differently across them.

### Medicine

Medicine supervisors’ knowing in practice was shaped by two main practice structures including: (1) consistent teams and (2) diverse and distributed clinical activities. Firstly, they worked with the same team (i.e., including advanced trainees/registrars, junior doctors and medical students) over several weeks. This meant that, compared to ED supervisors, they could set expectations and then monitor their performance over long periods of time.

Secondly, in internal medicine, their days and weeks were punctuated by diverse clinical activities including clinics; handover meetings; consultations; meetings; administrative work; and ward rounds. To complete these activities, the team worked in distributed ways. This meant trainees did not always directly work with supervisors. Yet supervisors ensured, despite being distributed, they were available using technology e.g. mobiles and messaging groups to provide guidance, for example. “The thing that I found most useful in communicating – cause I’m off the ward so much in clinics – is we have a messenger group, and we just text” (P12 Medicine). Another approach used the hospital’s electronic medical records to monitor the ward round virtually. P4 Medicine explains, “… even when the registrar and the resident are doing the ward round together, I will be sitting in my office reading the notes and disagreeing with things…and reading them out going, no, you shouldn’t do that.”

Supervisors valued ward rounds as an opportunity for trainee development. It afforded them opportunities to work directly with trainees and observe them in action. It was also when supervisors directly taught trainees:

…when I’m on the ward [round] team, that is a perfect time to teach, right - because you have the attention of your team; you’re stuck with them for a couple of hours every day, and you teach them things on the bedside, and you teach them with real life patients and real life scenarios and so it’s always a better environment to learn and a better way for them to appreciate things. (P1 Medicine)

Yet, direct teaching was not the only strategy reportedly being used to facilitate trainee learning. As ward rounds comprised of discrete tasks e.g., consulting with patients, making decisions and writing up medical notes, the supervisors, also, created learning opportunities from these tasks. Central to their approach was ensuring that trainees were engaging in these tasks in ways aligned with them becoming doctors:

So, a lot of the time, interns feel their job is to just clerk and follow [up] the request forms … [I] tell them you [intern] are an important member of the team….I ask them like in a difficult patient,…what do you think we should do now? We’ve done everything and sort of asking everybody to just stop and think about it. That generates sort of a sense of belonging to the plan of the patient, not just a typing skill. (P2 Medicine)

Another strategy reported was that supervisors assisted trainees to develop efficiencies in completing routine tasks (e.g., writing notes; ordering laboratory tests). When trainees developed these capacities, supervisors then prioritised engaging them in thinking and acting that had the effect of advancing their capacities beyond undertaking these routine tasks. Supervisory knowing in practice manifested here is a process of adapting to the learner as well the developing trainees based on the requirements of the workplace (i.e. needing to complete routine tasks):

… it makes it easier to let them [trainees] do more if they’re organised. If they have all of the [order] forms there on the ward round, we can do the scripts [prescriptions] as we go. We can do the forms as we go. We can get everything quicker. Then we do more teaching. We do more delivery. And that makes it easier to teach. Yeah, that’s probably the main thing. If they’re organised, they have more time than - an organised junior doctor is invaluable. (P12 Medicine)

As well as developing routinised task-related capacities, all medical supervisors reported aiming to develop trainees’ abilities to lead ward rounds. A reported approach was switching roles amongst team members. For example, P2 asked “the resident to take over as the main doctor walking in the [patient] room” (P2 Medicine) while carefully judging the best time for this to occur e.g. less acute patient, is for this - “it’s usually like day three of the patient or something of being in hospital” (P2 Medicine). This approach, P8 elaborates:

It’s affording them the doing it part of learning and then allowing them to reflect on that with less acute cases and by - or clinically less risky patients - so that they are not overwhelmed by anxiety of causing harm, but so that they have the - they’re in the comfortable frame of mind where they can explore their clinician identity a bit more but also come back and get some feedback on what they’ve done. (P8 Medicine)

The overall expectation was that the trainees’ capabilities should improve over time and require less explicit guidance. In this approach, trainees’ progress was measured by the trainees asking less questions and requiring less prompting:

Yeah, we expect to see progression over the term and any of the residents I expect over the term that they’re going to gain a bit more autonomy, to the point that they’re comfortable doing their own ward rounds and comfortable making decisions without having to phone up the registrar or myself about prescribing something. (P4 Medicine)

For the medicine supervisors, practice structures, including sustained co-working with trainees, meant that their supervisory knowing in practice included setting expectations, assessing and monitoring trainee readiness over time, drawing on a variety of pedagogical practices (e.g., questioning, guide trainees to develop efficiencies and navigate practice, role switching; solo rounds with proximal guidance). Also, electronic technology afforded opportunities to augment trainee learning through supervision remotely.

### Surgery

As surgical supervisors engage in team-based practices similar to those of medical teams (e.g., registrar, trainee), commonalities existed in their pedagogical practices including getting to know trainees, setting expectations and ensuring that trainees were connected to key team members. Whilst the ward round was a key practice, surgical supervisors discussed this less as a learning opportunity. Indeed, within days of commencing their term, they might make a preliminary assessment of trainees’ readiness and the trainees were often expected to lead the ward round without direct supervision. The following quote illustrates this approach:

For instance, the JHO [junior house officer] …they start with me on Monday. They do the ward round themselves on the weekends after it…They are a bit thrown in the deep end and I acknowledge that. But they are watched throughout that week. They are trained - and I supervise that. I have conversations throughout that week to prepare them for that ward round. Normally it’s not a problem anyway. But I just have to figure out how they go. I get a phone call after the ward round on Saturday and Sunday every weekend. I very quickly see how they travel. (P5 Surgery)

This approach is distinct from the supervisory strategies enacted in surgical theatre, which were far more guided. Supervisors emphasised incremental assessment of trainees’ readiness and then assigning tasks and engaging trainees based on this assessment. All surgical supervisors found it challenging and took a long time to accomplish this supervisory practice. This was because operating is highly complex and risky, in terms of patient safety, and requires focused, intense and co-ordinated activities with high levels of shared understanding. Supervisory strategies, consequently, included: (1) priming trainees; (2) ‘handing over the scalpel’; and (3) participating in other aspects of theatre practice. These are briefly elaborated now.

Firstly, supervisors prepared trainees for theatre by talking through the operation, asking them to do preparatory reading and priming them on the complexity of the operation (e.g., if complex then there will be less time for discussion and questions and the trainees should save their questions until the end [one participant had a whiteboard in theatre for that purpose]). If the operation was going to be highly complex, they may ask trainees to go to another theatre. P5 explains his approach:

… If you cross a structure that is important to know then I might ask the team what’s that. Then you have a bit of teaching session about what’s that. Why is it important. … But it’s all - it’s again on the go. You have - during an operation you have some steps. They are very crucial to get them right. So, then it might be a bit more concentration involved on that part…then you don’t teach as much. But around that before and after there is room for teaching. (P5 Surgery)

Secondly, in terms of ‘handing over the scalpel’, supervisors reported progressing cautiously as the risks to patient safety are particularly high and multiple factors informed their decision including trainee readiness (i.e. confidence; procedural skills), ongoing assessment of trainee readiness (e.g., recognising the fragility of readiness in high stakes situations) and risk to self (e.g. the likely emotional toll for them). P19 elaborates on the complexity associated with this act of handing over the scalpel:

The hardest thing was to teach someone to operate…I am letting people, juniors, operate, but it can be tricky…you’re responsible for the patient and you have to understand the skill set of the [person] you are letting, so you’re handing the scalpel across, this is your patient, you’re handing the scalpel to another person, so you might know them, but they could have a bad day and things could go wrong and they are junior. So, you’ve got to train them to be able to do what you do just as efficiently and remember we’re training here. So, they don’t start out experts, but they’ve got to become experts. It’s nerve wracking, it’s difficult and it takes a lot, which is why some people don’t teach. They talk you through an operation, but they’ll never let you do it. (P19 Surgery)

For other surgical supervisors, focusing on operating was not the only way trainees could be engaged. For instance, whilst time in theatre is ‘prime time’ and highly sought after, simply having trainees in theatre without an opportunity to ever engage in a purposeful way was not considered to be a valuable or effective learning experience. Rather ‘doing’ (i.e. trainees engaging in goal-directed activities) was reported as being highly valued in surgery.

For surgical supervisors, the diverse practice structures, including ward rounds and operating, illustrate how decisions were made about the perspective complexity of the practice, this then varied the degree to which trainees’ readiness were assessed. For such complex and risky practices, much more scaffolding was used e.g., priming, observing and initially engaging trainees in low-risk tasks.

### Supervisory knowing in practice: from balancing to climbing

Throughout the analysis of the dataset, supervisors described their knowing in practice as a balancing act between patient care and clinical supervision: “I am not going to spend too much time teaching, because we just need to get stuff done.” (P15 ED). Yet, the examples above demonstrate how situational factors, combined with supervisors’ skills, were adapted to the particular learner and in situ and this influenced how supervisory knowing in practice was manifested in different specialities. Consequently, seemingly personal-specific supervisory practices emerged through this interplay.

Coming back to the rock-climbing metaphor, our supervisors were assessing the practice (i.e. rockface) e.g. for busyness or operation complexity, orientating the trainees to the practice (i.e. rockface) and assessing their trainees’ readiness for the climb and identifying pedagogic practices (i.e. handholds) to develop trainees’ capabilities. Many were connecting trainees’ past understandings of being a doctor with being a doctor in their particular practice. As the practice conditions changed (e.g. the rockface became more complex or the weather more inclement), supervisors adjusted their approaches accordingly.

The following example illustrates this complex interplay using the rock-climbing metaphor. Firstly, when the workload became hectic, P4’s supervisory knowing in practice manifested by ensuring their trainee was safe and that an appropriate handhold (i.e. safety points), such as debriefing about a challenging situation, was in place and then continuing the climb:

…there was one particular intern…who…had a particularly bad day where one patient was dying and the wife couldn’t accept it, somebody else wasn’t happy that the mum was dying as well and the poor intern was in the office in tears. So, I came around and established what was wrong and went to speak to both families and then went back to her and encouraged her that she’s doing a good job. Those kinds of days you’re not going to get much teaching in and it is a matter of making sure that your team is coping. (P4 Medicine)

Supervisors were clearly orchestrating complex facilitation of learning within the exigencies of clinical care on a daily basis, but their own emphasis seemed to be on *balancing* didactic teaching *and* patient care, as opposed to seeing the learning opportunities *embedded in* patient care. This emphasis on *balancing* seemed to obscure the learning facilitation they were affording their trainees.

## Discussion

Our study examined supervisors’ knowing in practice as they facilitated trainee learning in three specialities within one hospital. Drawing on interdependent learning theory (Billett, 2006) and practice theory (Gherardi, 2019) we found two common domains of supervisory practice existed across specialities: (1) Orientating and assessing trainees’ readiness to practice and (2) sequencing and enriching learning. Yet through the lens of practice theory, we identified that important differences in supervisory practices exist across three specialities. In their differences we identified supervisory knowing in practice as dynamic and shaped by: (i) disciplinary practices, (ii) situational requirements and (iii) clinician preference. More broadly, we have found that supervisory knowledge emerges from the context of its production and was anchored by (and in) supports in practice (Gherardi, 2019).

By examining supervisory knowing in practice and identifying the factors shaping it, our findings point to ways we can intertwine key research-informed insights to better under how supervisory knowledge is manifested in different specialities. Firstly, our findings aligned to others, in that different manifestations of supervisory knowledge are shaped by individual supervisor preferences and experiences (Goldszmidt, et al., 2015) and their distinct conceptualisations of supervision (Stenfors-Hayes, et al., 2010; Strand, et al., 2015). However, clinician preference alone did not shape their enactment of supervision. Equally evident was that their supervisory practices were being shaped by supervisors’ disciplinary practices and its unique affordances in time and place (i.e. situational requirements), and then based on their assessment (which was ongoing) of trainee readiness, they generated opportunities for trainee learning. These decisions were dynamic, and this finding resonates with the fluid supervisory approaches i.e. adjusting their approach depending on context identified by Gingerich et al. (2018). Whilst the pedagogical strategies, such as sequencing learning, also identified by Chen et al. (2015), came into play, what has been advanced here is that different affordances for sequencing were evident in different specialities. For example, sequencing was shaped by differing timeframes for co-working, responsibilities and team structures. Thirdly, because of a combination of situational requirements and disciplinary practices, certain learning activities could be planned for (e.g. surgery and medicine knew which trainees were coming) whilst others could not (e.g. ED supervisors did not know who they were working with on their shift). This finding is important as it alerts us to fresh ways of understanding what constitutes clinical supervision and the need to accommodate situational requirements, such as contextual factors as illuminated by Bates et al. (2016, 2018). Overall, supervisors from these three specialities enacted supervisory knowing in practice in distinct ways as informed by the realities of their specialities clinical processes and the specific circumstances of their enactment, including trainee experience and readiness. It was these factors that extended to the choice of pedagogic practices they adopted to facilitate trainee learning.

Whilst others have identified that clinical teaching is embedded within and responds to the clinical environment within internal medicine (Steinert, et al., 2017), we show the adaptation of supervisory enactments in relation to practices in different specialities. Our findings also reinforce the work of Cantillon et al. (2020) who, when exploring clinical supervisor identity, found that clinical supervision is highly situated and shaped by sociocultural contexts. Whilst our work, which focussed on knowing (i.e. the enactment of supervisory knowledge) and goes beyond being (i.e. identity), further confirms the situatedness of supervisory practice by showing how supervisory knowledge emerges in the doing (i.e. enactment). Similarly, researchers have shown how feedback practices are shaped by and shape specialty feedback cultures (Bearman et al., 2023). Combined these findings emphasise that clinical supervision is not wholly a cognitive act whereby generic supervisory skills are applied to practice (Irby, 1994). What has been advanced here is that supervisory practice is participatory, and supervisors come to know what is possible through the combination of situational requirements and disciplinary practice. This means that effective supervision is going to look different in different specialities (Billett, 2001; Gherardi, 2001). Indeed, what is acceptable in one practice may not be considered acceptable in another. For instance, from our findings supervisors from different specialities engaged trainees in different ways on ward rounds. These findings suggest that how clinical supervisors’ skills are developed cannot be wholly reliant on faculty development programs sitting outside of practice. Instead, supervisory ways of knowing are a product of practice, and we cannot reshape supervision without attending to clinical practice.

This study, by introducing the concept of supervisory knowing in practice, offers a way to reconceptualise clinical supervision, that is, it is situated in and shaped by practice and supervisors’ ways of knowing emerge from the practice. Acknowledging that new conceptual perspectives can take time to integrate, the explanatory metaphor of rock climbing (whilst not derived from our findings rather it helped explain them) (Gherardi, 2019), offers a helpful way to make sense of this reconceptualisation of clinical supervision (Lingard & Goldszmidt, 2019). In particular, the rock-climbing metaphor helps reframe the problem - not as working and learning being in opposition but in understanding how these are brought together in situ/deftly by supervisors.

The rock-climbing metaphor has potential to contribute to clinical supervision in several ways. Firstly, when made explicit, trainees and supervisors may benefit from a shared understandings about the enactment of supervisory practice within their discipline. For instance, discussions based on the metaphor will likely help trainees to appreciate why the supervisor are doing things in a particular way. Secondly, the dynamic nature of supervisory knowing in practice, captured by the metaphor, offers a helpful way for clinical supervisor to re-conceptualise and talk about supervision and its complexities beyond the notion of ‘balancing teaching and practice’. For these supervisors, the notion of ‘balancing’ seemed to be constraining their understandings of what they were accomplishing and yet they persisted with the ‘balancing goal’. Finally, the rock-climbing metaphor reminds us that we can teach the skills that will work well in a climbing gym, but in practice it might be raining or the rockface may be more challenging than expected. It is important that faculty development programs attend to practice to illuminate how supervision can be accomplished within different settings, different specialities and with different trainees. In other words, supervisors can attune to reading the rockface and identifying handholds. This is about understanding and exploring practice in-depth.

Overall, our findings suggest that the following considerations will be helpful to advancing conceptualisations of effective clinical supervision. Firstly, we need to remove and reconcile the distinctions between clinical supervision and patient care. One way to achieve this is to embed supervision within clinical care activities. Embedding supervision means that supervision needs to be tailored to and accommodate the exigencies and circumstances of clinical practices. As clinical practices are shaped by both how medical specialties are enacted and the specific circumstances of their enactment, this should be central to informing how supervision is enacted and the strategies selected to support trainee learning. Perhaps this will lead to more effective supervision and supervisors will have not to choose between patient care or clinical supervision.

### Strengths, limitations and future research

This study explored supervisory knowing in practice in three specialty groups. The study’s strengths included its comprehensive theory informed design and data analysis. The synergy between interdependent learning theory (Billett, 2006) and knowing in practice (Gherardi, 2019) helped illuminate the moment-by-moment acts of facilitating trainee learning. Secondly, our findings are supported by rich descriptions to improve transferability and our sample was diverse.

We acknowledge the limitations of our sampling being confined to a single health service. Whilst our interviewees were able to elaborate on their practice through stories and exemplars, further research using observation can add further nuance and detail to unpacking practice. Alternatively, using a practice-based study approach – “interview to the double” (Gherardi, 2019, p. 338) where the interviewer asks the interviewee to imagine they need to walk (i.e. instruct) an alternative (or double) through their practice. As the interviewee describes their practice, they are asked to talk through how they should behave. Further research using video-reflexive ethnography (VRE)(Iedema, et al., 2019; Iedema, et al., 2013) may offer a way to illuminate and assist with this change. Importantly, our subsequent VRE work (Noble, et al., 2019), will focus on exploring assessing trainees’ readiness different specialities from perspective of trainees and supervisors and examine processes of sequencing and enriching learning through day-to-day practice. However, other strategies such as longitudinal audio diaries (Monrouxe, 2009) may allow the nuances of supervisory knowing in practice, related to (i) disciplinary practices, (ii) situational requirements and (iii) clinician preference, to be unpacked day to day over time.

The notion of ‘balance’ initially informed our study and indeed was how our supervisors framed supervisory skills. Adopting the notion of supervisory knowing in practice will require a reframing of supervision. Whilst the metaphor of rock-climbing has offered a new way to explore clinical supervision and account for practice, it does have some limitations. For instance, supervising from afar and use of technology does not fully align to the rock-climbing metaphor.

## Conclusion

We found that clinical supervision as a practice is shaped to different degrees by: (i) the disciplinary practices (i.e. specialty), (ii) the situational manifestation of that practice and (iii) the preferences of the supervisors, which together influences how supervisors facilitate their trainees’ learning. Supervisory knowing in practice and the metaphor of rock climbing offer a new way of conceptualising clinical supervision with implications for more situated approaches to faculty development.

### Supplementary material: interview guide

*Let’s discuss your role as a senior clinician*:

- What is your clinical speciality?
- What does a usual day look like for you (how many patients; trainees; inpatients/outpatients/theatre).
- How is your medical team structured?
- What roles do you have? How do you juggle these various responsibilities?
- How do you see your role as educator?

*Let’s discuss how you engage with and work with trainees (i.e. medical students or junior doctors) to support their learning*:

- When you have a new trainee in the team, how are they introduced to the work activities? What about training/education activities/expectations?
- Can you please provide examples of trainees’ usual jobs/tasks and how you help them to learn from doing these jobs?
- Which workplace activities or tasks do you find most useful to support junior doctor/student learning? How do you used these to their full advantage?
  - How do these differ for medical students and junior doctors?
- How do you support trainees’ development across a term? Examples.
- What factors assist you in developing trainees?
- What factors hamper your ability to develop trainees? E.g. workload; trainees’ approach to work; own ability as a supervisor.
- When developing trainees, how do you engage patients in this process?

*Let’s discuss how you balance clinical work whilst developing trainees*:

- How do you manage caring for your patients and trainee education? (or how do you balance clinical commitments with supporting junior trainees’ development?)
- Can you give me an example from the last week where you had to juggle patient care and trainee education/support their learning? What happened? How did you manage it? Which did you prioritise? Why? (prompt for specific teaching moments with patients e.g. ward rounds, seeing patients, end of term assessments (or other work-based assessments, feedback episodes etc.).
- What happens to trainee development when there are high levels of clinical commitments? What when clinical commitments are less?
- Can you give me an example where you were able to prioritise trainees’ learning despite a heavy clinical workload?
- How do others in the department balance clinical commitments with trainee development?

*Concluding questions*:

- If you could change one thing to make it easier for you to support the learning of junior trainees’ whilst balancing patient care commitments, what would it be?
- What has been the most significant experience you have had when supporting junior trainees’ learning through practice?
